# Differences in the Pulsatile Component of the Skin Hemodynamic Response to Verbal Fluency Tasks in the Forehead and the Fingertip

**DOI:** 10.1038/srep20978

**Published:** 2016-02-24

**Authors:** Toshimitsu Takahashi, Yoriko Takikawa, Reiko Kawagoe

**Affiliations:** 1 Department of Neurophysiology, Juntendo University Graduate School of Medicine, Tokyo, 113-8421, Japan; 2 Dynamic Brain Network Laboratory, Graduate School of Frontier Biosciences, Osaka University, Osaka 565-0871, Japan; 3 Department of Brain Physiology, Graduate School of Medicine, Osaka University, Osaka 565-0871, Japan

## Abstract

Several studies have claimed that hemodynamic signals measured by near-infrared spectroscopy (NIRS) on the forehead exhibit different patterns during a verbal fluency task (VFT) in various psychiatric disorders, whereas many studies have noted that NIRS signals can reflect task-related changes in skin blood flow. If such a task-related skin hemodynamic response is also observed in the fingertip, a simpler biomarker may be developed. Furthermore, determining the difference in the response pattern may provide physiological insights into the condition. We found that the magnitude of the pulsatile component in skin hemodynamic signals increased on the forehead (p < 0.001 for N = 50, p = 0.073 for N = 8) but decreased on the fingertip (p < 0.001, N = 8) during the VFT, whereas the rate in both areas increased (p < 0.02, N = 8). We also did not find a repetition effect in both the rate and the magnitude on the fingertip, whereas the effect was present in the magnitude (p < 0.02, N = 8) but not in the rate on the forehead. These results suggest that the skin vasomotor system in the forehead could have a different vessel mechanism to psychological tasks compared to the fingertip.

A verbal fluency task (VFT) is a neuropsychological test that assesses cognitive function in the frontal cortex. In a VFT, participants are asked to generate as many words as possible within a specified time limit in response to a letter or a semantic cue^1^. Several studies in the psychiatric field have claimed that cerebral blood flow signals, as measured by near-infrared spectroscopy (NIRS) on the forehead, showed significantly different response patterns for various psychiatric disorders when patients performed a VFT^2^,^3^,^4^,^5^,^6^,^7^,^8^,^9^,^10^,^11^,^12^,^13^. Recently, several researchers have inferred that the different aspects of frontal NIRS signaling during the VFT may reflect decreased frontal brain function in the patient and used this finding to diagnose patients with depression^6^,^7^,^13^,^14^.

However, recent studies have noted that the NIRS response signals could reflect task-related changes in skin blood flow, to a greater or lesser extent^15^,^16^,^17^,^18^,^19^,^20^,^21^,^22^,^23^,^24^,^25^,^26^,^27^. In particular, Takahashi and colleagues reported that a change in the NIRS signal (ΔoxyHb) was strongly correlated with the magnitude of the pulsatile component in skin blood flow signal on the forehead when simultaneously measured with a laser Doppler tissue blood flowmeter (R^2^ = 0.92). This finding suggested that the NIRS response to the VFT could highly correlate with autonomic activities^15^. This result may predict that hemodynamic responses in the skin vasomotor system in the forehead during VFT are modulated by certain mental demands that affect the autonomic system. Furthermore, if such a task-related skin hemodynamic response is also observed in the fingertip, then a simpler biomarker may be developed.

Interestingly, however, in the study by Takahashi and colleagues^15^, the skin blood flow signal on the forehead positively responded to the VFT, whereas it is generally known that skin blood flow on various hairless body parts, such as the hand, decreases because of increasing sympathetic activity^28^, including because of a mental arithmetic task^29^. It is physiologically notable to determine whether the sympathetic skin vasomotor system in the forehead has a special mechanism that is sensitive to mental factors and, subsequently, can be used to diagnose particular psychiatric disorders. Furthermore, detecting this response in other body parts also demonstrates that the hemodynamic response is sensitive to such mental factors apart from its polarity.

In this study, to examine the differences in autonomic skin hemodynamic responses to the VFT on between the forehead and other body parts, we focused on pulsatile components involved in the skin hemodynamic signals that were simultaneously measured on the forehead and the fingertip during a VFT that was used in a previous study^15^. In this experiment, the hemodynamic signals on the fingertip were recorded with a pulse oximeter, whereas those on the forehead were recorded with a laser Doppler flowmeter. As it is not appropriate to directly compare two signals acquired by different methods, we first extracted the pulsatile component from each raw signal using time-frequency analysis and then assessed whether the signals increased or decreased compared to the baseline and whether signal changes occurred between the task blocks for each measurement site. Here, we assumed that the signal change in blood flow in a local tissue region correlated with that in blood volume (see Discussion).

## Methods

### Participants

We analyzed data from 8 of the 50 healthy volunteers who had participated in our previous study (7 males and 1 female; age 19 to 25 years, median = 20.5 years)^15^. All participants were neurologically normal and naive to the task. The study was approved by the review boards of Juntendo University and was conducted in accordance with the approved guidelines. All participants provided written informed consent before participating.

### Tasks

The detail of the task design of the VFT was described in our previous study^15^, which was nearly the same as the task used by Kameyama *et al*. (2006)^7^ except that word generation was repeated twice in two blocks. Each block consisted of a 60-s-long word generation period that was preceded by 30 s and followed by 70 s with control periods (See Fig. 1 in Takahashi *et al*., 2011^15^). During the word generation period, a recorded voice announced three different syllables, one-by-one, with an interval of 20 s. The participants had to generate and pronounce as many words (non-proper nouns) as possible in the 20-s period that started with each syllable. During the control periods, participants were instructed to repeat five syllables at a rate of approximately one syllable per second.

### Data measurements

In our previous study, we recorded hemodynamic signals on the left fingertip by pulse oximeter (photoplethysmograph output; Model 3012, Nonin Medical Inc., USA) during the VFT with NIRS and Doppler signals in only 8 of 50 participants. Ambient temperature was maintained at approximately 22 °C using the air conditioner to avoid the effect of temperature change on skin blood flow^29^. Detailed methods of the NIRS and Doppler signal measurements from the forehead were described in the study^15^. Briefly, we placed eleven sources and eleven detector NIRS optodes (FOIRE-3000, Shimadzu Corp., Japan) in a matrix of 2 × 11 to cover the frontal pole and bilateral fronto-temporal regions with a separation distance of 30 mm. The lower row of the matrix was positioned along the Fp1–Fp2 line according to the international 10/20 system. We then placed a laser Doppler tissue blood flow meter probe (FLO-C1, Omegawave Incorporated, Japan) on the left or right forehead below the location of F7 or F8. The Doppler tissue blood flow meter collected data from the scalp layer within 1 mm from the probe^21^, and the analog output was recorded simultaneously by the NIRS system.

### Data analysis

We compared the task response of pulsatile signal components involved in Doppler signals on the forehead to those involved in the pulse oximeter signals on the fingertip to examine whether autonomic activity induced by task demand causes a different vascular dynamic.

All signals were analyzed using MATLAB software (MathWorks Inc., USA). The pulsatile component involved in a signal was estimated by time-frequency analysis using the short-time Fourier transform algorithm (spectrogram function in MATLAB) after resampling at 10 Hz without an alias effect.

**Figure 1:**
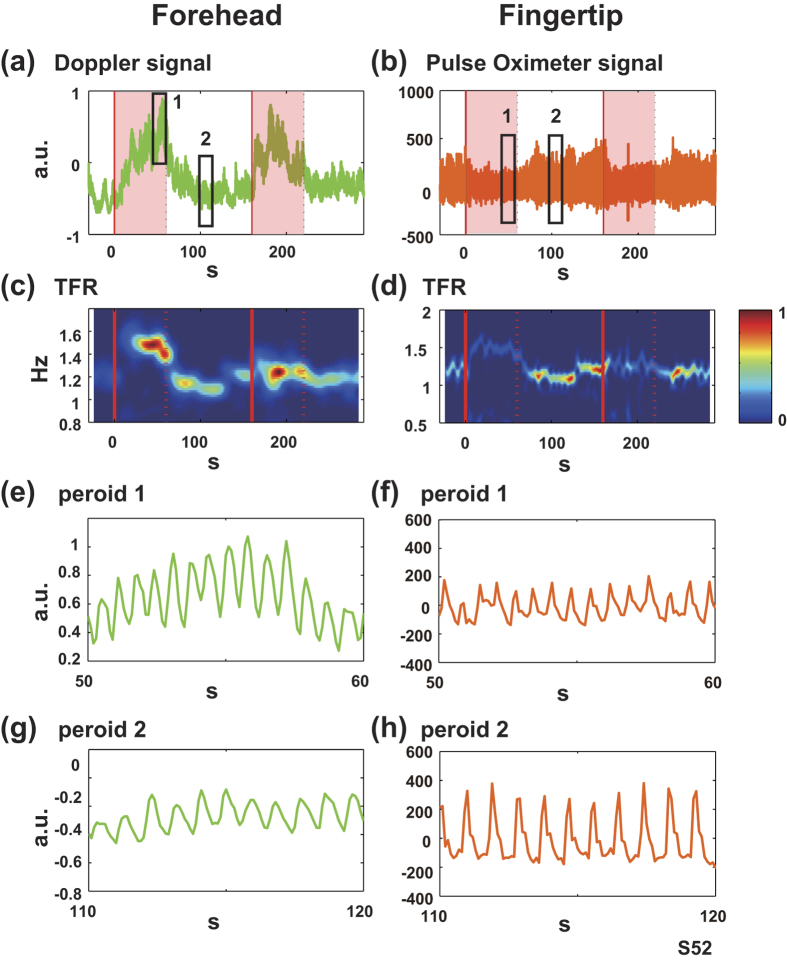
Typical examples of pulsatile components of Doppler signals on the forehead (left panels) and pulse oximeter signals on the fingertip (right panels). (**a,b**) Raw signals of skin blood flow measured by a laser Doppler tissue blood flow meter and by pulse oximetry during VFT (arbitrary unit). The task blocks were successively repeated two times and consisted of control (non-shaded) and word generation (shaded) periods. (**c,d**) Time-frequency representation (TFR) plots of Doppler signals (**a**) and pulse oximeter signals (**b**), respectively. Normalized amplitude of PSD was represented in pseudocolor. (**e,g**) Enlarged waveforms in periods 1 and 2 in (**a**). Both the magnitude and the rate of the pulsatile component were larger during the word-generation period. (**f,h**) Enlarged waveforms in periods 1 and 2 in (**b**). The rate of the pulsatile component was also larger, whereas the magnitude was smaller during the word-generation period, which was opposite to the signals on the forehead.

Examples of time-frequency representation (TFR) of Doppler signals and pulse oximeter signals are shown in Fig. 1c,d. First, we defined the rate time series of the pulsatile component by choosing the frequency that had the maximum power spectral density (PSD) value at each time point, ranging from 0.5 to 2 Hz on a TRF. The time series of maximum PSD values chosen above was defined as the magnitude time series of the pulsatile component. Then, we segmented the pulsatile rate and the magnitude time series into two task blocks and converted each of them to percent signal changes (baseline was 10 sec and occurred before the onset of the word generation period). We then examined whether there was a significant change in the task-response signal averaged over the period from 25 to 35 seconds after the task onset (Wilcoxon rank test). We further investigated the relationships between changes in the task-response signal in the first block and the second block (Wilcoxon signed rank test).

## Results

### Task response of the pulsatile components

Figure 1 shows typical examples of the task response of the pulsatile components that are involved in the Doppler signal on the forehead and the pulse oximeter signal on the fingertip from a single participant. In the TFR of the Doppler signal (Fig. 1c), the rate and magnitude of the pulsatile component in the Doppler signal on the forehead skin generally increased during the word generation period in comparison with the control period (Fig. 1e,g), as reported in our previous study^15^. Nevertheless, in regards to the pulsatile component in the pulse oximeter signal on the fingertip skin, the magnitude decreased during the word generation period in comparison with the control period while the rate increased (Fig. 1f,h).

**Figure 2:**
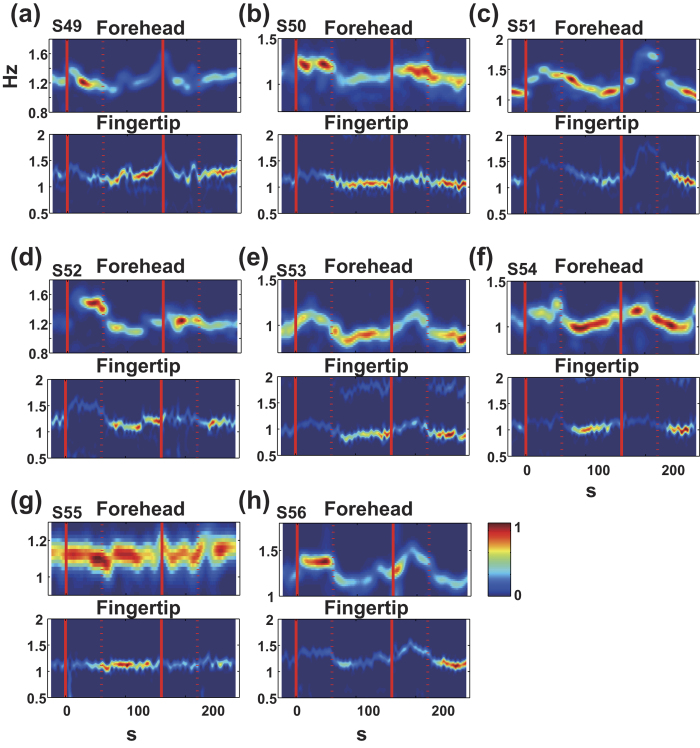
TFR profiles for all participants (N = 8). The top panel shows TFR for the forehead signals, and the bottom panel shows the TFR for the fingertip signals in all participants (**a–h**). The TFR patterns of all participants were almost consistent with the typical pattern that is shown in (**c**) and (**d**) in Fig. 1.

As shown in Fig. 2, the pattern of TFRs was basically consistent in all participants. The magnitude of the pulsatile component (defined as the maximum PSD value at each time point on TFR) tended to increase during the word generation period on the forehead but decreased on the fingertips whereas the rate (frequency) of the pulsatile component increased both on the forehead and on the fingertip.

**Figure 3:**
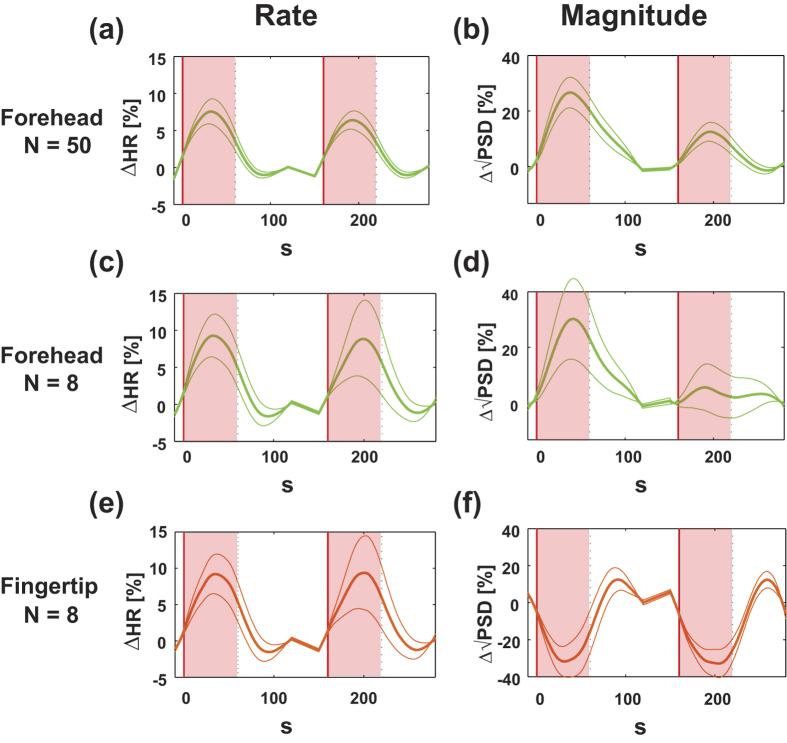
Comparison of the frequency and magnitude of the pulsatile components in the forehead and the fingertip. The rate of the pulsatile component increased during the word generation period both on the forehead and on the fingertip (**c,e**). During word generation, the magnitude on the forehead increased, whereas the magnitude decreased on the fingertip (**d,e**). The repetition effect was only shown in the magnitude of the pulsatile component in the Doppler signal. (**a,b**) Large population data of Doppler signals (N = 50) adapted from Takahashi and colleagues^15^ for comparison.

This observation can be clearly summarized in signal percent change plots, as seen in Fig. 3. The rate of the pulsatile component increased during the word generation period in comparison with the control period on both the forehead and the fingertip (Fig. 3a,c,e. p < 0.02). Yet, the magnitude of the pulsatile component on the forehead clearly increased during word generation (Fig. 3b,d, p < 0.001 for N = 50, p = 0.073 for N = 8), whereas the magnitude on the fingertip decreased (Fig. 3f, p < 0.001).

### Repetition effect

Furthermore, significant signal changes in the pulsatile rate on the fingertip or the forehead between the two task blocks were not observed (p > 0.15 on the forehead: Fig. 3a,c; p = 0.94 on the fingertip: Fig. 3e). Yet, the signal change in the magnitude on the forehead in the second block significantly decreased compared with the first blocks (called ‘repetition effect’) (p < 0.02, Fig. 3b,d). There was no effect on the fingertip (p = 0.74, Fig. 3f).

## Discussion

In the present study, we clearly demonstrated differences in autonomic skin hemodynamic responses to the VFT between the forehead and the fingertip by simultaneous measurement.

We found that during VFT, the magnitude of the pulsatile component in observed signals of skin hemodynamics increased on the forehead but decreased on the fingertips, whereas the rate increased both on the forehead and on the fingertip.

The increase in pulsatile rate could be explained as a reflection of sympathetic activity that was caused by the task demand. The decrease in pulsatile magnitude on the fingertips is consistent with the previous reports that skin blood flow on the fingertip decreases during mental stress tasks, such as mental arithmetic^29^. It is well known that in most skin areas except the head, an increased sympathetic activity causes decreased blood flow that is mediated mainly through α-adrenoceptors distributed on the smooth muscle cells of blood vessels^28^,^30^,^31^. The VFT may be a task in which participants feel mental stress. Therefore, sympathetic activity could occur during the task and result in a decrease in the magnitude of the pulsatile component on the fingertip. In contrast, the magnitude of the pulsatile component on the forehead clearly increased during the task in the present study. These opposing hemodynamic response patterns suggest that mental stress can induce sympathetic vasodilator and/or vasoconstrictor mechanisms according to the body part. Regarding the facial skin, vasodilation and vasoconstriction responses to sympathetic stimulation are different in facial regions, but the physiological mechanism responding to mental stress is not yet understood^32^. A previous study reported that sympathetic vasodilator responses on the forehead are evoked by arousal stimuli and mental stress at normal room temperatures, but this effect varies across individuals^33^. Drummond investigated the physiological mechanism of the sympathetic vascular response on the forehead using a pharmacological method and reported the distribution of β-adrenoceptors on the skin vasculature on the forehead^31^,^34^. He also reported cases in which the beta blockade prevented increases in blood flow during mental arithmetic^35^. In the present study, however, it was unclear whether the increase in the magnitude of the pulsatile component on the forehead was caused by active sympathetic vasodilation mediated by β-adrenoceptors or was passively reflected by an increased cardiac output that resulted from weakness or the absence of sympathetic vasoconstriction via α-adrenoceptors.

Takahashi and colleagues reported that the NIRS signal on the forehead during VFT with successive task blocks had a repetition effect and was strongly correlated with the magnitude of the pulsatile component in skin blood flow signal on the forehead in 50 healthy volunteers^15^. They suggested that the possible mechanisms include local autonomic vasodilation or systemic changes in the stroke volume. In the present study, in contrast to the forehead, we found that the magnitude of the pulsatile component on the fingertip and the rate of the pulsatile component on both the two areas showed no repetition effect during VFT. This result may support the possibility that task-related hemodynamic changes in the forehead during VFT are controlled by a local autonomic vasodilation mechanism that is different from the autonomic control over the heart.

The limitations of this study include the differences in the vascular response measurement methods between the two areas, that is, the hemodynamic signals on the fingertip were recorded by the pulse oximeter (photoplethysmograph output), which detects local blood volume, whereas those on the forehead were recorded by laser Doppler flowmeter reflected local blood flow. We assumed in this study that task change in blood flow in a local tissue region would correlate with blood volume, based upon the “windkessel model,” which described the flow-volume dynamics of the arterial system^36^. According to the framework, a change in the local blood flow leads to a change in the local blood volume after the change in the flow is integrated over time^37^. Indeed, in the previous study, the Doppler signal was correlated closely with the NIRS signal, which reflects local blood volume, after the Doppler signal was integrated with a certain time constant, when both signals were recorded simultaneously on the forehead during VFT^15^. Moreover, it has reported that changes in finger blood flow was directly paralleled by the size of the finger pulse volume in healthy participants using plethysmography^38^.

An additional limitation is that only the hemodynamic response on the fingertip was examined as a comparison to that on the forehead. It may include difficulty generalizing a description of the overall skin blood control because it has been known that skin blood vessels on fingers have unique structural and functional features (high-density arteriovenous anastomoses) that contribute to thermoregulation, as well as those on toes and tips of nose and ears^39^. Accordingly, to describe unique aspects of the skin hemodynamic response on the forehead during VFT, the skin hemodynamic response on various anatomical positions needs to be examined. Nevertheless, it is noteworthy that skin vasculature in a different anatomical position from the forehead also showed hemodynamic response to the VFT, as this finding strongly supports the evidence in the previous study that autonomic vascular response may influence NIRS measurements on the forehead during the VFT^15^.

For clinical application to the diagnosis of psychiatric disorders, it is important to reveal the physiological mechanisms of these hemodynamic responses in the forehead skin vasculature and the other areas to several types of cognitive demands and their relationship to cerebral blood flow responses in NIRS signals.

## Conclusions

We found that during VFT, the magnitude of the pulsatile component in observed signals of skin hemodynamics increased on the forehead but decreased on the fingertips, whereas the rate increased on both the forehead and the fingertip. Additionally, we did not find a repetition effect in either the rate or the magnitude of the pulsatile component on the fingertip, whereas the effect existed in the magnitude but not in the rate on the forehead. These results suggest that the vasomotor system in the forehead skin could have a different sympathetic vascular response mechanism to mental stress tasks compared to that in the fingertip.

## Additional Information

**How to cite this article**: Takahashi, T. *et al*. Differences in the Pulsatile Component of the Skin Hemodynamic Response to Verbal Fluency Tasks in the Forehead and the Fingertip. *Sci. Rep.* **6**, 20978; doi: 10.1038/srep20978 (2016).
